# A recurrent adaptive mutation in the transmembrane 2B protein of an insect picorna-like virus in a nonnative host

**DOI:** 10.1128/jvi.01239-25

**Published:** 2025-12-22

**Authors:** Oscar M. Lezcano, Lara Fuhrmann, Reinder T. Bos, Haitao Wang, Milou Stevens, Niko Beerenwinkel, Martijn A. Huynen, Ronald P. van Rij

**Affiliations:** 1 Department of Medical Microbiology, Radboud University Medical Center 6034, Nijmegen, the Netherlands; 2 Department of Biosystems Science and Engineering, ETH Zurich 211122 , Basel, Switzerland; 3 SIB Swiss Institute of Bioinformatics, Basel, Switzerland; 4 Key laboratory of Food Quality and Safety of Jiangsu Province, Institute of Plant Protection, Jiangsu Academy of Agricultural Sciences 117941, Nanjing, China; 5 Department of Medical BioSciences, Radboud University Medical Center 6034, Nijmegen, the Netherlands; Wageningen University & Research, Wageningen, Netherlands

**Keywords:** insect immunity, RNA virus, *Dicistroviridae*, *Picornavirales*, *Drosophila melanogaster*, virus evolution, virus-host interactions, cross-species transmission

## Abstract

Virus emergence is often due to cross-species transmission and adaptation to the new host. We studied the effect of innate immune responses on shaping virus populations in native and nonnative virus-host combinations, using as a model system *Drosophila melanogaster* infected with either Drosophila C virus (DCV) or cricket paralysis virus (CrPV). In this host, the cGAS-like receptor 1 senses viral double-stranded RNA and produces cyclic dinucleotides (CDNs) to activate the STING protein and induce an antiviral response. Both viruses were serially passaged in three host conditions differing in their cGAS-STING response: wild-type (WT) flies, *Sting* knock-out (KO) flies, and flies with a primed immune response by CDN injection. We found no immune-related effects on virus evolution, but we uncovered the CrPV nonstructural 2B protein as a key regulator of cross-species transmission. Nucleotide diversity specifically accumulated in the *2B* gene during passage of CrPV in its nonnative *Drosophila* host, while *2B* of the fly-adapted DCV displayed markedly lower and constant nucleotide diversity. In particular, the CrPV 2B D29N variant was selected in all six virus lineages evolved in WT and *Sting* KO flies, with an estimated selection coefficient greater than 0.2. This variant replicated faster and was more lethal than the parental virus in all three host backgrounds. 2B is a predicted transmembrane protein, which we found to be associated with cellular endomembranes and may be involved in replication organelle formation. Our findings suggest a role for the 2B protein in adaptation to a new host independent of the cGAS-STING pathway.

**IMPORTANCE:**

The forces driving virus evolution are central to understanding cross-species transmission and virus emergence. It is well established that the adaptive immune system drives virus evolution in mammals, but whether innate responses likewise drive virus evolution upon host shifts is less well understood. In this manuscript, we used *Drosophila melanogaster* as a model to study the evolution of a native and a nonnative pathogen under conditions in which innate antiviral immunity is either abolished or enhanced. Using an experimental evolution approach, we find little evidence for adaptive evolution of the natural pathogen Drosophila C virus. In contrast, we observed a recurrent adaptive mutation in the viral nonstructural 2B protein in the nonnative cricket paralysis virus, independent of the antiviral cGAS/STING pathway. Our work provides insights into viral adaptation to new hosts and the characteristics of the 2B protein of dicistroviruses, a family comprising important model insect viruses.

## INTRODUCTION

Emerging viral diseases are often the consequence of cross-species transmission. Host shifting is restricted by the phylogenetic distance between the hosts and often requires molecular adaptation to unrecognized host factors such as entry receptors and the immune system of the new host (1). Indeed, host shifts appear to occur most frequently among RNA viruses (2), likely due to their error-prone replication and adaptability. Also, within natural hosts, the immune system is an important driver of virus-host coevolution, which is especially well established for the adaptive immune response of mammals. Models suggest that immune memory selects new surface antigens unrecognized by the host immune system, forcing the virus into uncharted antigenic space (3). Other simulations proposed that the immune response accelerates viral evolution during persistent infections (4). Indeed, in hepatitis C virus and human immunodeficiency virus-1 (HIV-1) infection, significant associations were observed between the emergence of specific viral mutants and specific human leukocyte antigen (HLA) types, likely a consequence of immune-driven selection of variants with decreased antigen-HLA binding affinity (5, 6). However, also in organisms without adaptive immunity, host resistance may drive the evolution of escape variants. This is exemplified by an experimental evolution study in *Escherichia coli*, where the bacteriophage λ repeatedly evolved the ability to use an alternative surface receptor in response to the evolution of host resistance due to reduced expression of the original LamB receptor (7).

In mammals, the main innate antiviral pathway is the interferon response (8), which can be activated by the cGAS-STING signaling pathway, an evolutionarily conserved pathway that originated in bacteria to control bacteriophage infections (9). The cGAS protein (cyclic GMP-AMP synthase) acts as a pattern recognition receptor that senses double-stranded DNA (dsDNA) in the cytosol and, in response, synthesizes cyclic di-nucleotides (CDNs), in particular 2′3′-cyclic GMP-AMP (cGAMP). CDNs are soluble molecules that act as second messengers, activating the conserved STING protein (stimulator of interferon genes). STING is an adapter protein embedded in the endoplasmic reticulum (ER). Upon activation, it translocates to the Golgi apparatus through an unknown mechanism that is essential for downstream signaling (reviewed in reference 10). This, in turn, leads to translocation of the transcription factor IRF3 (interferon regulatory factor 3) into the nucleus and the expression of type I interferons and interferon-stimulated genes.

Despite lacking the interferon response, insects have a functional cGAS-STING pathway, albeit with marked differences compared to the mammalian pathway. For instance, *Drosophila melanogaster* encodes two cGAS-like receptors (cGLRs) capable of producing CDNs (11, 12), and different *Drosophila* species may even encode up to seven cGLRs (13). Of these, cGLR1 is activated by dsRNA, an intermediate in RNA virus replication and a hallmark of RNA virus infection (13). cGLR1 then produces 3′2′-cGAMP, a strong STING agonist that activates *Sting*-dependent signaling, leading to the translocation of the NF-ΚB transcription factor Relish to the nucleus and the transcriptional activation of STING-regulated genes (SRGs) (14). This response has been shown to limit viral RNA accumulation and extend the survival of flies infected with several viruses, including the dicistroviruses Drosophila C virus (DCV) and cricket paralysis virus (CrPV) (15), both positive-sense RNA viruses from the genus *Cripavirus* (*Dicistroviridae*).

Here, we aimed to gain a quantitative understanding of the specific effects of host shifts and innate immune adaptation on virus evolution. We performed an experimental evolution study of a native and a nonnative RNA virus in three *D*. *melanogaster* lines, differing in the activity of the cGAS-STING pathway. Along with the natural fly pathogen DCV, we used the nonnative CrPV, originally isolated from Australian field crickets (*Teleogryllus oceanicus* and *Teleogryllus commodus*) (16). While CrPV has also been found in metagenomic data from Australian honey bees (17), it has to the best of our knowledge never been reported in wild-caught flies. We passaged the viruses for 10 fly generations, keeping 3 parallel lineages per virus and condition, after which the evolved lineages were characterized by RNA-sequencing. We found limited immune-related effects on virus evolution but observed high genetic diversity in the CrPV *2B* gene. In particular, the conserved aspartic acid (D) at position 29 was substituted by an asparagine (N) in all virus lineages evolved in wild-type (WT) and *Sting* null mutant flies. We found that the CrPV D29N variant replicated faster and was more virulent than the WT CrPV strain in all fly conditions (virulence defined here as the capacity to cause host damage [18]). Our results suggest that viruses may not readily evolve resistance to cGAS/STING immunity and highlight the importance of the nonstructural 2B protein in viral adaptation to a non-native host species.

## RESULTS

### Experimental CrPV and DCV evolution

**Fig 1:**
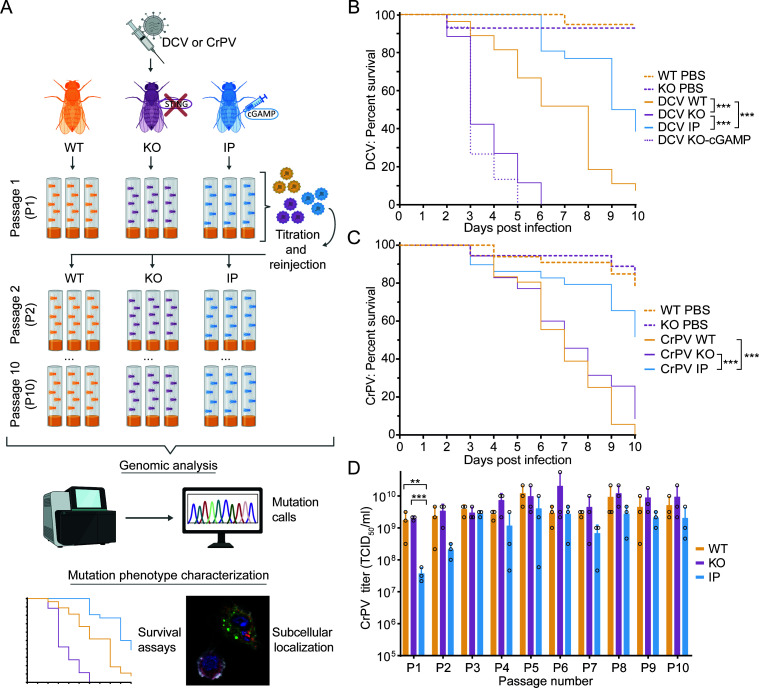
Experimental evolution of CrPV and DCV in *Drosophila melanogaster* with differing cGAS-STING response. (**A**) DCV or CrPV parental stocks were serially passaged in three distinct fly conditions: WT, *Sting* knockout (KO), and cGAMP immune primed (IP). This process was carried out with three virus lineages per condition for 10 fly generations. Flies were intrathoracically inoculated, and viral titers were determined after each passage. The evolved virus populations were subjected to genomic analysis using next-generation sequencing, and the phenotype of the fixed mutations was studied. (**B and C**) Survival curves of WT, *Sting* KO, and IP flies infected with either 100 TCID_50_ of DCV (**B**) or 1,000 TCID_50_ of CrPV (**C**) (*n* = 40 flies per condition). Phosphate-buffered saline was taken along as a mock infection control. As an additional control, cGAMP was co-injected with DCV in *Sting* KO flies in (**B**). Log-rank test *P*-values are indicated with asterisks: ***P < 0.0001. (**D**) CrPV titers in lysates of flies after each passage of all evolving lineages. Bars represent the means and SD of the three replicate viral lineages. Individual data points are shown as circles. Bonferroni adjusted *P*-values of the estimated marginal means test are indicated with asterisks: **P < 0.01, ***P < 0.001.

To investigate the influence of the cGAS-STING pathway on host adaptation and virus evolution, we experimentally evolved DCV and CrPV in flies differing in their immune response: WT flies (*w^1118^*, hereafter WT), *Sting* null mutant flies generated by CRISPR/Cas9 editing (*dSTING^L76GfsTer11^* [15], hereafter knockout [KO]), and WT flies whose immune response was ectopically activated by injection of 3′2′-cGAMP (hereafter referred to as immune primed [IP]) (Fig. 1A). As anticipated (13–15), modulation of cGAS-STING pathway activity correlated with fly survival after DCV infection. *Sting* KO flies were more sensitive to infection than WT flies (*P* < 0.0001, log-rank test; mean survival times 6.7 days for WT and 3.7 days for KO), and IP flies were less sensitive than WT flies (*P* < 0.0001, log-rank test; mean survival time of 8.8 days for IP flies; Fig. 1B). The protective effect of cGAMP injection was *Sting*-dependent as cGAMP injection in *Sting* KO flies did not affect survival after DCV infection (Fig. 1B, KO-cGAMP), thereby also excluding that a possible direct interaction between cGAMP and the virus inoculum caused the protective effect of cGAMP in WT flies. Likewise, cGAMP injection extended fly lifespan after CrPV infection, although *Sting* inactivation did not affect survival (*P* < 0.001, log-rank test; mean survival times of 6.8, 7.2, and 8.7 days for WT, KO, and IP, respectively; Fig. 1C).

We allowed virus populations to evolve during 10 fly generations (passages), with three independent lineages per host condition (Fig. 1A). The DCV parental stock was a host-adapted strain generated by serial dilution of the virus evolved in WT flies for 10 passages from our previous study (19). The CrPV parental stock was generated from an infectious molecular clone (20). Virus titers were measured after each passage to standardize the inoculum for subsequent passages and to minimize population bottlenecks and differences in the rate of genetic drift. Viral titers of CrPV and DCV after each passage generally showed small differences between fly conditions across most passages (CrPV: Fig. 1D; DCV: Fig. S1A), likely due to virus titers reaching a plateau at this time point (3 days post-infection). The exception was the first passage of CrPV evolution, where the viral titers in IP flies were significantly lower than in WT and KO flies (61-fold and 71-fold, respectively; *P*-adj <0.005, estimated marginal means for pairwise comparison; Fig. 1D). In fact, viral titers were barely high enough to inoculate the next generation with the desired 1,000 TCID_50_ inoculum, and we therefore decided to increase the CrPV inoculum to 5,000 TCID_50_ from passage three onwards for all lineages.

### Increasing DCV synonymous nucleotide diversity

**Fig 2:**
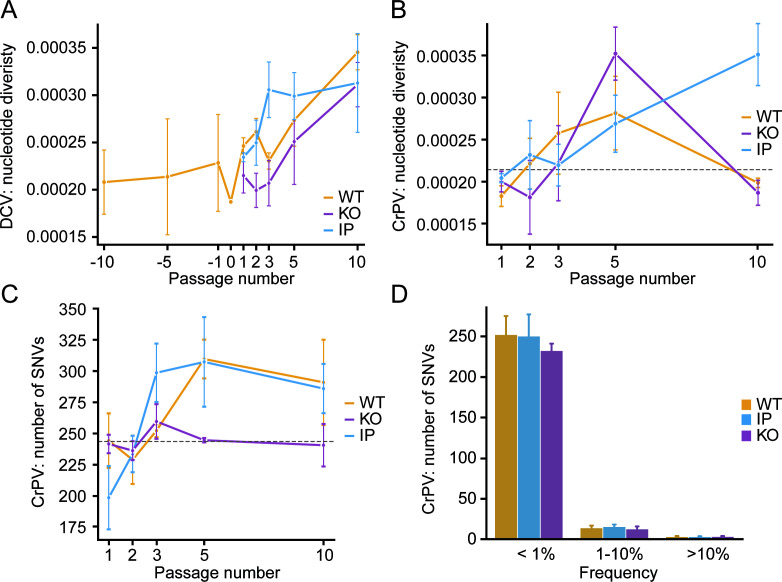
Host condition-specific diversity trends in viral populations during experimental evolution. (**A**) Mean nucleotide diversity of evolved DCV populations. Diversity values from DCV evolved in WT flies from our previous study (19) are integrated and denoted as passages –10, –5, and −1. Passage 0 is the parental stock, and passages 1–10 are the evolved DCV populations of the current study. Data are shown as means and standard errors across the three replicate lineages for each genotype. (**B–C**) The mean nucleotide diversity (**B**) and number of SNVs (**C**) of evolved CrPV populations from WT, *Sting* KO, and IP flies across passages. Data are shown as means and SEM across the three replicate lineages for each genotype. The dashed lines mark the number of SNVs and mean nucleotide diversity in the parental CrPV stock. (**D**) Frequency distribution of SNVs for CrPV virus populations from the indicated host conditions across all passages and replicates. Bars indicate means, and error bars indicate 95% confidence intervals based on the bootstrap distribution.

We studied molecular evolution during the experiment by RNA-sequencing at passages 1, 2, 3, 5, and 10 and computed viral nucleotide diversity across passages. We obtained a mean read depth across the genomes of 47,501 and 29,539 for the DCV and CrPV samples, respectively, which was consistent across samples (Fig. S2). For DCV populations evolved in WT flies, we incorporated the diversity values from our previous study (19). As expected, serial dilution used to prepare the parental virus (P0) reduced nucleotide diversity, consistent with population bottlenecking (Fig. 2A). By weighing nucleotide diversity per site according to the fraction of possible synonymous or non-synonymous substitutions, we found that the serial dilution specifically reduced non-synonymous nucleotide diversity (Fig. S1C). Afterward, we observed a significant increase in nucleotide diversity as passages progressed across all DCV populations, regardless of their host condition (Fig. 2A, *P* < 0.0001, linear mixed model, Table S1). Increasing nucleotide diversity was predominantly explained by the accumulation of synonymous single-nucleotide variants (SNVs) (Fig. S1B through D). Only a single SNV in the 3′ untranslated region accumulated in all lineages, but this SNV preexisted in the parental virus. However, as we observed no indications for adaptive DCV evolution during the experiment, such as the condition-specific fixation of SNVs or the accumulation of nucleotide diversity, we focused our subsequent analyses on the evolved CrPV populations.

### Transient increase and subsequent collapse of CrPV nucleotide diversity

In the CrPV parental stock, we identified a total of 244 SNVs with respect to the consensus sequence of its 9.2 kb genome, of which four occurred at frequencies above 10% (Fig. S3A). As the parental stock was produced from an infectious clone, these SNVs must have accumulated during *in vitro* transcription or the 48 hours of replication in *Drosophila* S2 cells used to prepare the stock.

As observed for DCV, nucleotide diversity significantly increased for CrPV with passage number (*P* < 0.001, estimate = 0.05, linear mixed model, Table S2). However, there was a significant decrease in diversity between passages 5 and 10 for populations evolved in WT and *Sting* KO flies (*P* = 0.001, estimate = −0.39, linear mixed model). A significant interaction was observed between the IP background and passage 10 (*P* = 0.01, estimate = 0.25, linear mixed model), indicating a sustained increase in nucleotide diversity of viral populations evolved in IP flies at later passages, in contrast with the sharp decline observed in WT and KO flies (Fig. 2B; Fig. S3B). At the same time, we observed increasing numbers of SNVs from passage 1 onwards in the populations evolved in WT and IP flies (Fig. 2C) with a mean of 244 and 199 SNVs at passage 1, respectively, which increased to a mean of 291 and 286 at passage 10. In contrast, for CrPV populations evolved in *Sting* KO flies, the mean number of SNVs remained roughly at the same level as the parental stock. The vast majority of SNVs occurred at low frequency (median: 0.07%, Fig. 2D), whereas only between 9 and 28 SNVs occurred at a frequency above 1%, and between 1 and 5 SNVs at a frequency above 10%, across all host conditions, passages, and replicates (Fig. 2D).

### Specific accumulation of nucleotide diversity in the CrPV *2B* and RdRp genes

**Fig 3:**
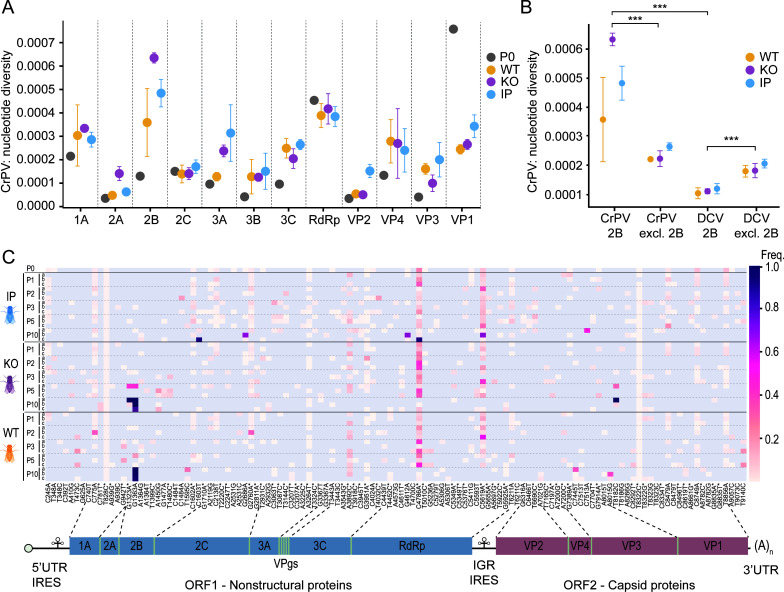
Nucleotide diversity accumulates specifically in the CrPV *2B* gene. (**A**) Nucleotide diversity for each CrPV gene. For each host background, the mean nucleotide diversity of the three replicate lineages over all passages was computed. Data are shown as means and standard errors across the three replicate lineages for each condition. (**B**) Nucleotide diversity in the viral *2B* gene and the remaining coding region in the evolved DCV and CrPV populations. Data are shown as means and standard errors across the three replicate lineages for each host background. Bonferroni adjusted *P*-values of the estimated marginal means test comparing the four categories are indicated with asterisks: ***P < 0.001. (**C**) Heatmap of SNV frequencies in CrPV populations evolved in WT, *Sting* KO, and IP flies across the viral genome. Lowercase letters a, b, and c indicate the three independent lineages. Non-variable sites are indicated in gray. Non-synonymous mutations are marked with an asterisk. Only SNVs with a frequency greater than 0.01 in at least two samples are shown. Lower panel: schematic representation of the CrPV genome. Gene coordinates follow UniProt annotation (Replicase polyprotein, Q9IJX4 and structural polyprotein, P13418); capsid protein names are according to ICTV (21), which differs from the P13418 UniProt entry. IRES, internal ribosome entry site; IGR, intergenic region.

We calculated the nucleotide diversity for each viral gene and each condition (Fig. 3A; Fig. S4) and found that the mean nucleotide diversity varied significantly between viral genes (*P*-adj. < 0.001, two-way mixed ANOVA), but not between host conditions (*P*-adj. = 0.202). Post hoc pairwise comparisons (Materials and Methods, Table S3) revealed that the *3D* gene encoding the RNA-dependent polymerase (RdRp) and the *2B* gene showed significantly increased diversity compared to seven and ten other genes, respectively (Table S3). In addition, *2B* was the only gene that showed higher diversity in the evolved populations across all three host conditions than in the parental stock (Fig. S4). Moreover, the diversity in *2B* was significantly higher compared to the remaining coding region (Fig. 3B, *P*-adj. = 0.0004, estimated marginal means with Tukey’s adjustment), indicating that the dynamics in *2B* dominate the diversity in the whole genome. Conversely, in evolved DCV populations, we observed the opposite trend: the diversity in *2B* was significantly lower than in the remaining coding region (Fig. 3B*, P-*adj. = 0.0024, estimated marginal means with Tukey’s adjustment). Furthermore, a direct comparison of *2B* diversity between DCV and CrPV populations revealed significantly lower diversity in DCV (Fig. 3B, *P*-adj. < 0.0001, estimated marginal means with Tukey’s adjustment), suggesting the importance of the *2B* gene in CrPV adaptation to the *Drosophila* host and stringent selection on *2B* in DCV.

### Independent fixation of CrPV 2B D29N in six lineages

A complex pattern of SNV retention and emergence was observed along the CrPV genome across different lineages and passages (Fig. 3C). Many SNVs present in the parental stock were retained in most lineages (e.g., C775A, T828C, C1602A, G2760A, A3264T, C4786A), while others showed temporal variations. For instance, C8749A and T8856A persisted until passage 5 but were absent in passage 10, and G4612A was retained only in populations from IP flies. We observed the *de novo* appearance of A3892C, a silent mutation of Arg154 in the 3C protease (AGA to CGA), in all host conditions across most passages and replicates at frequencies of 2.0%–14.9%. Another interesting *de novo* SNV was G1363A, resulting in an aspartic acid to asparagine substitution at position 29 of the 2B protein (D29N). This substitution emerged at passage 2 in WT flies for the first time at a frequency of 0.03% and in *Sting* KO flies at passage 3 at a frequency of 0.05%. In all replicate lineages in WT and *Sting* KO flies, this mutation was fixed at passage 10 at frequencies above 82%. In contrast, in viral populations from IP flies, G1363A was only detectable in one replicate lineage at a frequency of 0.2% at passage 3, but rather than increasing in frequency, it became extinct in the following passages. In one of the lineages from *Sting* KO flies (KO-a), we detected an additional synonymous mutation, G1323A, in close proximity to and at similar frequencies as the G1363A substitution. Local haplotype analysis confirmed the co-occurrence of G1363A and G1323A in a high proportion of reads that carry at least one of those mutations at passages 5 and 10 (2101/2501 and 4792/4802, respectively), suggesting that G1323A is a hitchhiking mutation.

### D29N causes a marked increase in viral virulence

**Fig 4:**
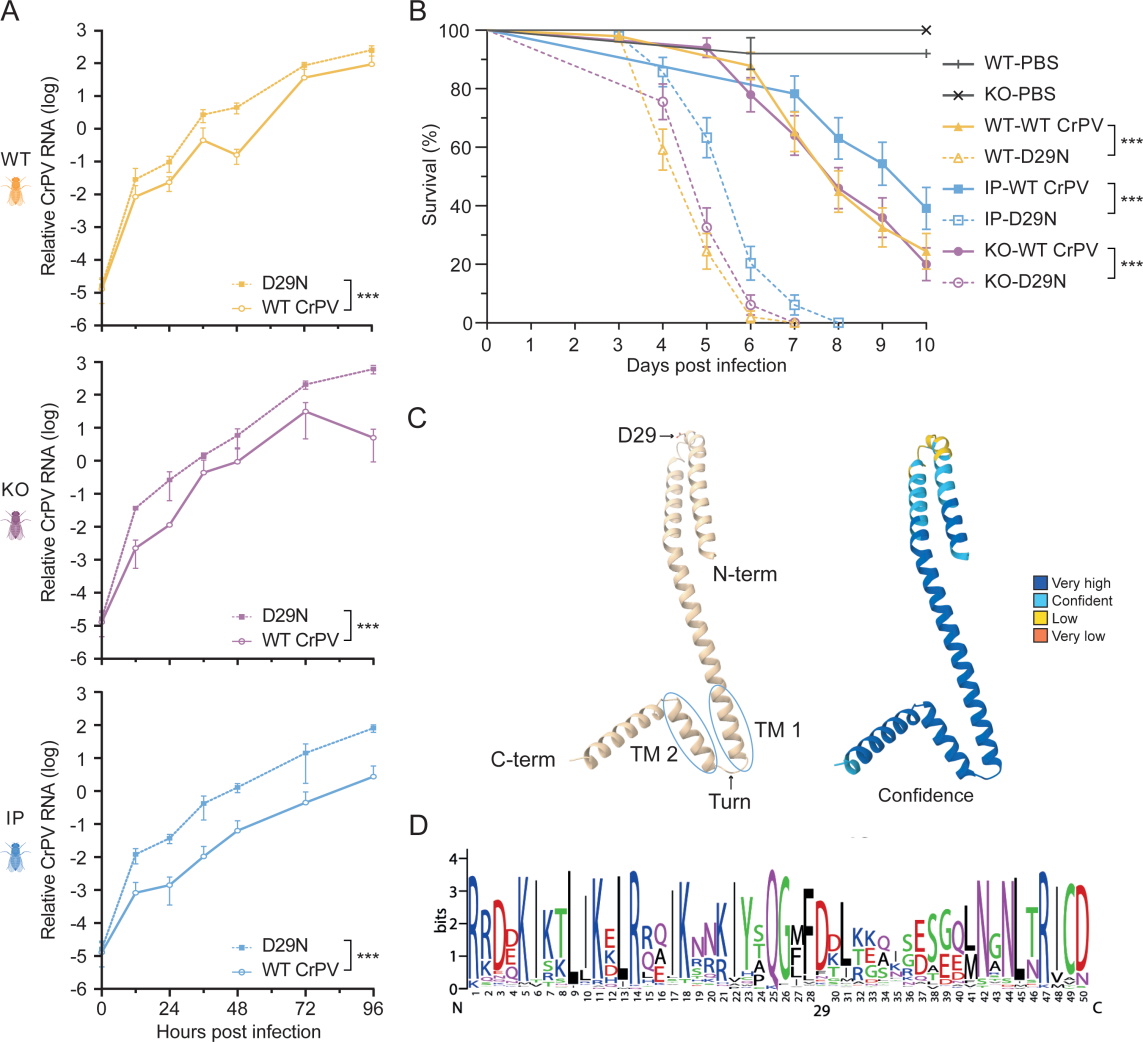
Higher replication kinetics and virulence of the CrPV D29N variant. (**A**) Growth curves of WT CrPV and D29N CrPV in WT, *Sting* KO, and IP flies inoculated with 1,000 TCID_50_. Three pools of five flies were harvested per time point, and viral RNA was quantified by RT-qPCR. The means and SD of CrPV RNA levels relative to *rp49* are shown. The difference between WT and D29N CrPV was statistically significant in a linear model (*P* < 2.75 x 10^−10^). (**B**) Survival curves of WT, *Sting* KO, and IP flies inoculated with PBS or 1,000 TCID_50_ of either WT or D29N mutant CrPV. Two- to five-day-old females (*n* = 50) were intrathoracically inoculated and monitored daily for survival. Log-rank test *P*-values are indicated with asterisks: ***P < 0.0001. (**C**) AlphaFold 3 prediction of the CrPV 2B structure as a monomer. The position of the D29 residue and both predicted transmembrane α-helices (TM) are indicated. Color coding in the right panel indicates the confidence level of the prediction: yellow, low confidence (70 > plDDT > 50); light blue, confident (90 > plDDT > 70); dark blue, very high confidence (plDDT > 90). (**D**) Sequence logo of the first 50 amino acids of the 2B protein and 131 homologous proteins identified by BLASTp.

The recurrent fixation of D29N in multiple independent lineages suggests that it confers an evolutionary benefit to the virus. To experimentally study the phenotype of D29N, we introduced the mutation into the infectious clone and compared the growth kinetics of WT CrPV and the D29N CrPV mutant in WT, *Sting* KO, and IP flies. The D29N mutation significantly increased viral RNA levels (*P* < 0.0001, linear mixed-effects model, Table S4), with an estimated 5- to 10-fold increase in all three host conditions compared to the WT virus (Fig. 4A). Stimulation of the cGAS-STING pathway by co-injection of 3′2′-cGAMP significantly reduced CrPV RNA levels by a magnitude of 26.7 for both variants in WT flies (*P* = 0.0014, linear mixed-effects model, Table S4), indicating that D29N did not affect sensitivity to cGAS/STING-mediated immunity. In line with the increased viral replication rate, survival of flies infected with the D29N mutant virus was significantly reduced compared to WT CrPV-infected flies in all host backgrounds (*P* < 0.0001, log-rank test; mean survival times of 8.2, 8.2, and 9.0 days for WT, KO, and IP flies infected with WT CrPV and 4.8, 5.1, and 5.7 for WT, KO, and IP infected with the CrPV D29N), although 3′2′-cGAMP co-injection still extended fly survival (Fig. 4B).

We approximated the selection coefficient of the D29N mutant relative to WT CrPV using two independent approaches: tracking changes in nucleotide frequencies during the experimental evolution study (Fig. 3C) and comparing the replication rates of WT and D29N mutant CrPV that we estimated assuming exponential growth (Fig. 4A, Materials and Methods). Both methods yielded similar results, with mean estimates of 0.287 and 0.267 for the frequency and RNA-based estimates, respectively, indicating increased fitness of the D29N mutant virus (Table S5). Overall, these data indicate that D29N causes an increase in viral replication and virulence.

### 2B is a predicted transmembrane protein that localizes to endomembranes

**Fig 5:**
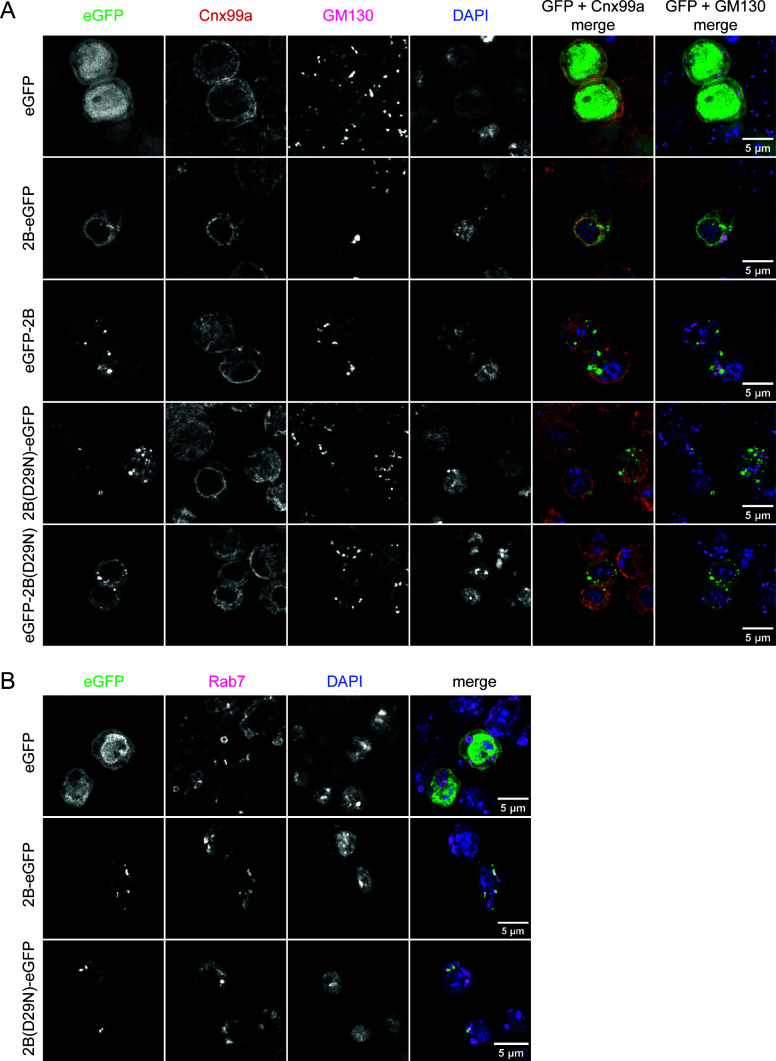
Subcellular localization of the WT and D29N CrPV 2B proteins. Confocal microscopy images of *Drosophila* S2 cells transfected with plasmids encoding the indicated proteins. Cells were fixed 48 h after transfection and stained with (**A**) antibodies against the ER protein Calnexin 99A (Cnx99a) and the *cis* Golgi protein GM130, and (**B**) antibodies against the lysosome protein Rab7 (magenta). Nuclei were stained with DAPI. In the merged images in (**A**), eGFP is shown in green, Cnx99a in red, GM130 in magenta, and DAPI in blue.

No experimental data exist about the function of CrPV 2B or any of its homologs, including DCV 2B that we identified using PSI-BLAST (22) or HHPred (23). Picornaviruses such as coxsackievirus and poliovirus (*Enterovirus* genus) also encode a protein named 2B that, as in CrPV, lies directly upstream of the 2C RNA helicase. To find evidence for homology between CrPV 2B and enterovirus 2B, we ran HHPred in the *pairwise profile alignment* mode, in which we compared CrPV 2B with the complete coxsackievirus A10 (CVA10) and poliovirus polyproteins. CrPV 2B specifically aligned with the poliovirus 2B protein and the CVA10 2B protein (Fig. S5A and B), albeit with high E-values (E = 0.55 and E = 0.24, respectively). The region that is most similar between the 2B proteins is a stretch of 49 amino acids that corresponds to the two predicted transmembrane (TM) helices in CrPV 2B (Fig. S5A) (24) and two experimentally determined TM helices in the coxsackievirus and poliovirus 2B proteins (25, 26). The D29 residue lies upstream of these helices and appears well conserved among homologs that could be detected with BLASTp (Fig. 4C). AlphaFold structure prediction (27), whose modeling accuracy improved by the addition of lipids, as well as HHpred (Fig. 4C; Fig. S5C), suggested that all three 2B proteins are predominantly α-helical. They likely adopt a helix-turn-helix motif that traverses organelle membranes and would expose D29 toward the cytoplasm (26). Based on its tentative homology with the enterovirus 2B proteins, we hypothesized that the CrPV 2B protein localizes in the endomembrane system of the host cell (25, 26). To experimentally validate this, we tagged the WT and D29N 2B proteins with eGFP at either the N or C terminus and studied their subcellular localization, using *Drosophila* proteins known to localize in the ER (Calnexin 99a), cis-Golgi (GM130), and lysosomes (Rab7) as markers (Fig. 5). We observed that 2B localized in the ER (red), but also showed punctate patterns corresponding to the cis-Golgi (Fig. 5A, magenta), lysosomes (Fig. 5B), and other membranous organelles, whose identity remains to be established. The D29N mutation did not seem to drastically affect the subcellular localization of the 2B protein.

**Fig 6:**
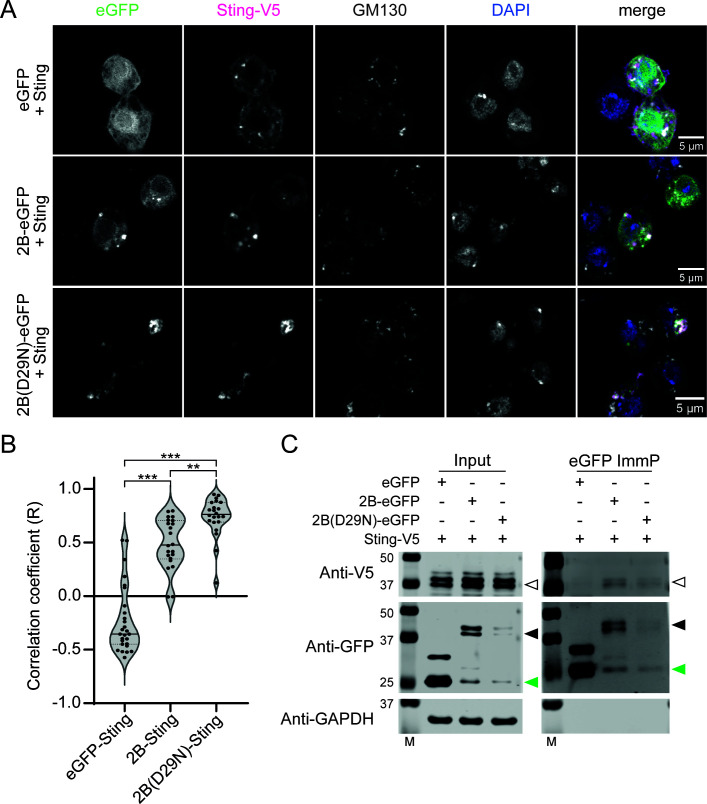
Sting colocalizes with the CrPV 2B protein. (**A**) Confocal microscopy images of *Drosophila* S2 cells transfected with a combination of the indicated plasmids. Cells were fixed 48 h after transfection and stained with antibodies against the V5 tag and the *cis* Golgi protein GM130. (**B**) Violin plot of Pearson’s correlation coefficient (R) of Sting with eGFP (*n* = 27), 2B-eGFP (*n* = 22), or 2B(D29N)-eGFP (*n* = 22) in individual cells, calculated in Fiji using the Costes method. The median is represented with a solid black line and the quartiles with dashed lines. Adjusted *P*-values from Tukey’s multiple comparison test are indicated with asterisks: **P < 0.01, ***P < 0.001. (**C**) Co-immunoprecipitation of Sting with 2B and 2B(D29N). S2 cells were transfected with the indicated plasmids, and the cell lysates (input) and eGFP immunoprecipitates (eGFP ImmP) were analyzed by western blot using eGFP, V5, and GAPDH antibodies. The white arrow heads indicate Sting-V5 (expected size 43.1 kDa), black arrow heads indicate 2B-eGFP and 2B(D29N)-eGFP (43.3 kDa), and green arrow heads indicate eGFP. As previously reported for human STING, *Drosophila* Sting showed distinct migrating bands, which might be due to post-translational modifications (28). M, size marker.

Considering that human STING is embedded into the ER membrane, we analyzed whether *Drosophila* Sting would interact with CrPV 2B. We epitope-tagged Sting and co-expressed the fusion construct with either WT or D29N 2B-GFP fusion proteins or an eGFP control in S2 cells. The punctate pattern of 2B was highly similar to the pattern produced by the Sting protein (Fig. 6A), which was validated by colocalization analysis (median Pearson’s correlation coefficient R = 0.48; Fig. 6B). Strikingly, an even stronger correlation was observed for the 2B D29N mutant (R = 0.76; Fig. 6B). The interaction between 2B and Sting was further validated by a co-immunoprecipitation assay. We found that the Sting protein was recovered in immunoprecipitations of both the WT and D29N 2B-GFP proteins, but did not coprecipitate with the eGFP control alone (Fig. 6C). Together, these data suggest that the 2B protein localizes in cellular endomembranes, where it interacts with Sting. However, given that the D29N mutation was also selected in flies lacking Sting, it is unlikely to represent an adaptation to optimize 2B interactions with Sting.

## DISCUSSION

In this study, we have explored the impact of the cGAS-STING pathway on virus evolution using *D. melanogaster* infected with DCV or CrPV as a model. We allowed the viruses to adapt to flies with a modulated cGAS-STING response, hypothesizing that variations in pathway activity would affect virus evolution at the genomic and phenotypic level. We found no effect of the STING pathway on DCV evolution, but instead noted a gradual increase in synonymous nucleotide diversity in all host conditions. Likewise, for CrPV, we found no evidence for adaptation that was specific to different cGAS-STING regimes. In contrast, we observed that nucleotide diversity accumulated especially in the CrPV *2B* gene in all conditions, while DCV exhibited reduced diversity in *2B* compared to the remaining coding regions, consistent with findings from our previous study (19). Specifically, for CrPV, we detected an SNV resulting in a D29N amino acid substitution in the 2B protein that was selected in all viral lineages from WT and *Sting* KO flies, while its frequency did not increase in viral populations from IP flies, despite being detected in one lineage at an early passage. CrPV mutants harboring D29N replicated faster and were more virulent than the WT virus in all host backgrounds. The appearance of parallel adaptive mutations driven by cross-species transmission has been previously observed in experimental evolution studies of DCV adapting to different *Drosophila* species (29) and vesicular stomatitis virus adapting to either human or dog cells (30). As *Drosophila* is not the natural host of CrPV, our data suggest that the 2B mutation likewise emerged to facilitate adaptation to its nonnative host.

We found that DCV nucleotide diversity continued to increase with passage number, suggesting that the virus populations had not reached equilibrium yet. Equilibrium is reached when the gain of genetic diversity is equal to the loss caused by purifying selection and random drift (31). This state seems difficult to attain in virus populations, as nucleotide diversity not only increases during previous relatively short-term evolutionary experiments (32–34) but also in long-term experiments, for example, HIV-1 experimental evolution over more than 3 years, during which mutations continue to accumulate (35). Likewise, the number of polymorphic sites also continued to accumulate in anellovirus over a period of 30 years in chronically infected patients (36). Therefore, the time scale necessary for a virus to achieve equilibrium is likely too long to be achieved in our experiment.

In 6 of the 18 virus lineages in our study, nucleotide diversity decreased from passage 5 to passage 10. A common factor of those virus populations was the fixation of the adaptive D29N mutation in the CrPV *2B* gene. We hypothesize that the diversity decrease was caused by a selective sweep (37), as previously observed during the fixation of drug resistance variants in HIV-1 (38). The observation that the D29N mutant was not selected in IP flies despite its higher replication rates across all host conditions might be explained by altered evolutionary dynamics due to a reduction of the viral effective population size upon the stimulation of the cGAS-STING pathway. Under these conditions, evolution is expected to be dominated by random drift rather than deterministic selection occurring under large population sizes (39, 40). While we attempted to estimate the variance effective population size from temporal changes in synonymous allele frequencies, our experiment involved selection effects and population fluctuations that violate key assumptions of standard effective population size estimation methods (41), making such estimates unreliable in our system (41–43). We did observe, nonetheless, a significantly reduced viral RNA load in IP flies, suggesting a smaller total population size, which may contribute to the observed evolutionary patterns.

It is currently unclear why the 2B mutation facilitated CrPV host adaptation. The function of the 2B protein of members of the *Dicistroviridae* family has not been studied. However, their corresponding relative positions in the viral genome, putative homology based on sequence-profile comparisons, and the location of (predicted) transmembrane regions suggest that CrPV 2B is homologous to the 2B protein of enteroviruses, although direct evidence is lacking. Enterovirus 2B is an ion channel-forming protein, typically 50–120 amino acids in length. It contains two hydrophobic regions that adopt a helix-turn-helix conformation, enabling insertion into cellular membranes (reviewed in reference 44). The protein also has at least one amphipathic α-helical structure that can oligomerize to form transmembrane hydrophilic pores (45, 46), possibly as a tetrameric complex (47–50). Enterovirus 2B has viroporin activity, which is associated with increased membrane permeabilization, disrupted calcium homeostasis, and induction of apoptosis and autophagy (51). In addition, enterovirus 2B is associated with viral replication organelles, thought to be derived from modified ER and Golgi apparatus membranes (52). We indeed observed localization of CrPV 2B in cellular endomembranes, but whether the protein is also a viroporin and how mutations in such a protein would facilitate host adaptation remains to be studied.

The 2B D29N mutation was selected in both WT and *Sting* KO flies, and the mutant conferred a growth advantage to CrPV also in *Sting* KO flies, indicating that 2B D29N-mediated host adaptation is independent of the cGAS-STING pathway. It was therefore rather unexpected to find that CrPV 2B colocalizes with Sting, and that the colocalization was even stronger for the D29N 2B mutant. While this conundrum awaits being solved, it is interesting to note that enterovirus 2B has been shown to affect STING localization in mammalian cells. Specifically, the 2B protein of rhinovirus was found to reduce Ca^2+^ levels in the ER, triggering the relocation of STING to the replication organelles (53), where it acts as a proviral factor to promote viral replication (28).

Overall, our work provides new insights into viral adaptation to a new host and the characteristics of the 2B protein of dicistroviruses, a family comprising important model viruses to dissect insect virus-host interactions.

## MATERIALS AND METHODS

### Fly strains and husbandry

Flies were maintained at 25°C in standard fly food. Eggs were bleached and treated with tetracycline as described before (54), and the absence of Nora virus, DCV, Drosophila X virus, CrPV, and *Wolbachia* was verified as described earlier (19). Flies containing the *dSTING^L76GfsTer11^* null allele (15) (referred to as *Sting* KO in this study) were a kind gift from Jean-Luc Imler (CNRS-Université de Strasbourg). *w^1118^* flies were used as WT, as the *Sting* KO flies had been isogenized in this genetic background according to the procedure described for cGLR1 and cGLR2 in reference 11. IP flies were generated by the intrathoracic injection of WT flies with 69 nL of a 1 µg/µL solution of c[A(2′,5′)pG(3′,5′)p] (3′2′-cGAMP) (BioLog). Two- to five-day-old female flies, likely mated as they were obtained from a vial containing unsorted flies, were used for the experimental evolution study, growth curves, and survival assays.

### Plasmids

The CrPV *2B* coding sequence was amplified by PCR from cDNA of infected *w^1118^* flies and cloned into fusion vectors expressing eGFP under control of the Actin promoter using In-Fusion cloning (TAKARA). For the construction of pAct-2B-eGFP plasmid, the *2B* sequence was amplified using forward primer 2B-eGFP-For (5′-GGTACCTACTAGTCCccaccATGCGCCGAGATGAGAAAATTTCAACCTT-3′), containing the Kozak sequence and the start codon, and reverse primer 2B-eGFP-Rev (5′-GCCCTTGCTCACCATggatccacctgatccgccTTGGGTTGTTGTCTGCAAAATTTTGT-3′) encoding a linker sequence (GGSGGS). The PCR product was cloned into the pAct-eGFP-C backbone linearized with primers vectorFor (5′-ATGGTGAGCAAGGGCGAG-3′) and vectorRev (5′-GGACTAGTAGGTACCCCGATCC-3′). For the construction of the pAct-eGFP-2B plasmid, the *2B* sequence was amplified using forward primer eGFP-2B-For (5′-GACGAGCTGTACAAGGGCGGATCAGGTGGATCCCGCCGAGATGAGAAAATTTCAACCTT-3′) encoding the linker peptide and reverse primer eGFP-2B-Rev (5′-AGCTCAGGCCTTAGAttaTTGGGTTGTTGTCTGCAAAATTTTGT-3′) containing a stop codon. The PCR product was cloned into the pAct-eGFP-N backbone linearized with primers vectorFor (5′-TCTAAGGCCTGAGCTCGCT-3′) and vectorRev (5′-CTTGTACAGCTCGTCCATGCC-3′). The mutant fusion constructs pAct-2B(D29N)-eGFP and pAct-eGFP-2B(D29N) were generated by site-directed mutagenesis by amplifying either pAct-2B-eGFP or pAct-eGFP-2B with primers For (5′-GGTTTCTTTAATGATCTCAAAGGAGCAAAAGG-3′) and Rev (5′-TAAAGAAACCTTGGGTGTAAATTCTGTTG-3′). The linearized PCR product was recombined using In-Fusion cloning (TAKARA).

The pCrPV(D29N) infectious clone was generated by amplifying the 2B(D29N) sequence from the plasmid pAct-2B(D29N)-eGFP with primers For (5′-GAGAAAATTTCAACCTTGATTAAGAAG-3′) and Rev (5′-AATCAAGGCGGCACGATAC-3′) and the backbone from pCrPV-3 (20) with primers For (5′-CGTGCCGCCTTGATTGTAAT-3′) and Rev (5′-GTTGAAATTTTCTCATCTCGGCG-3′) using In-Fusion cloning (TAKARA).

The *Sting* coding sequence was amplified by PCR from cDNA of *w^1118^* flies and cloned into pAc5.1-V5-His-A (Invitrogen) using In-Fusion cloning (TAKARA) to produce Sting-V5. The forward primer (5′-GATCGGGGTACCTACccaccATGGCAATCGCTAGCAACGT-3′) contained the Kozak sequence, and the reverse primer (5′-AGGGATAGGCTTACCggatccacctgatccgccGTTGGAAATTTCGTCAATAGTTTTGGTTTTGTTT-3′) contained a sequence encoding a linker peptide (GGSGGS).

### *In vitro* transcription and transfection of the CrPV infectious clone

The plasmid encoding the CrPV molecular clone (pCrPV-3 [20]) was a kind gift from Eric Jan (University of British Columbia). *In vitro* transcription and transfection were performed as previously described (20). Briefly, the plasmids were amplified in Stellar-competent *E. coli*, purified, linearized with Ecl136II (Invitrogen), and used for *in vitro* transcription at 30°C for 3 h using the RiboMAX kit (Promega). The RNA was treated with DNase RG1 for 15 min at 37°C and purified using RNeasy columns (Qiagen). The viral RNA was transfected into *Drosophila* S2 cells (Invitrogen) in six-well plates containing 2 mL of Schneider’s medium. After 48 h, the supernatant was collected, centrifuged at 300* × g* for 5  min, aliquoted, titered, and stored at −80°C.

### Cells and virus stocks

The parental DCV and CrPV stocks were produced in *Drosophila* S2 cells (Invitrogen), which were maintained in Schneider’s medium supplemented with 10% fetal calf serum (Sigma) and 50 U/mL penicillin and 50 µg/mL streptomycin (Gibco) at 27°C. DCV previously passaged in WT flies for 10 generations (19) was bottlenecked by serial dilution in a 24-well plate, selecting the highest dilution that still showed cytopathic effects to inoculate a T25 flask of confluent S2 cells containing 7 mL of medium. After 72 h, the supernatant was collected, centrifuged at 300* × g* for 5 min, aliquoted, and stored at −80°C. Titers were determined by end-point dilution in 96-well plates, as described before (54), and expressed as median tissue culture infectious dose (TCID_50_), calculated using the Reed–Muench method.

### Experimental evolution

Thirty female flies of each host condition were intrathoracically inoculated with 1,000 TCID_50_ of DCV or CrPV in 69 nL of PBS, pH 7.3, using a Nanoject II microinjector (Drummond Scientific). For IP flies, 3′2′-cGAMP was co-injected with the virus inoculum into WT flies. Three lineages per host condition were used as independent replicates. For the first passage, flies were inoculated with the respective parental stock, and from P1 onwards, all 18 lineages were kept independently (nine lineages per virus). After each passage, pools of 10 flies were snap-frozen and homogenized two times for 10 s in 220  µL of PBS, using 1 mm silica beads in a Precellys homogenizer, centrifuged at 16,000* × g* for 10  min to discard fly debris, and the supernatant was transferred to a fresh tube, aliquoted, stored at −80°C, and directly titrated. The next generation of flies was then inoculated through intrathoracic inoculation. In the case of CrPV, the virus inoculum was increased to 5,000 TCID_50_ from passage three onwards, as the titers after the first and second passage were barely high enough to reach the desired inoculum for the next passage in the IP flies. For RNA extraction and preparation of a next-generation sequencing (NGS) library of the parental stock, 1 mL of TRI Reagent (Sigma) was added to 100 µL of virus stock, while for the viral lineages, pools of five flies were homogenized in 1 mL of TRI Reagent (Sigma) using 1  mm silica beads in a Precellys homogenizer. RNA was isolated according to the manufacturer’s instructions.

### Virus infections and survival assays

To study virus growth kinetics, 100 TCID_50_ of DCV or 1,000 TCID_50_ of WT or D29N CrPV were injected into 2- to 5-day-old WT, *Sting* KO, and IP flies. For the IP condition, 3′2′-cGAMP was co-injected with the virus into WT flies. Three pools of five flies per condition were harvested at the indicated time points and processed for RT-qPCR. To study virulence, the same dose and procedure were applied to 50 flies per condition. Survival was monitored daily and analyzed using the Kaplan-Meier estimator. The difference between the curves was compared using the log-rank test in GraphPad Prism 10.

### RT-qPCR

RT-qPCR was performed as previously described (19), using primers CrPVFor (5′-ACGAGGAAGCAACTCAAGG-3′) and CrPVRev (5′-GAGCCCGCTGAGATGTAAAG-3′) for CrPV RNA quantification.

### NGS library preparation

Total RNA from passages 1, 2, 3, 5, and 10 was extracted using TRi Reagent (Sigma) and reverse transcribed with Superscript IV (Thermo Fisher) and oligo(dT) primers. Viral cDNA was amplified in four overlapping amplicons of about 2.2 kb. The primers used for CrPV were as follows:

For1, 5′-CTCCCCGTGAGAAACCTTGTTT-3′;

Rev1, 5′-GTGTTTGTAAGCGTCGGGTTTG-3′;

For2, 5′-GATCCCGGACCGAGACATTG-3′;

Rev2, 5′-TTACCGCCTGACCAACCTTG-3′;

For3, 5′-ACGGATATGCTTGCCCCTTAAC-3′;

Rev3, 5′-TGGGGTGAAACATAGGGAATTCTC-3′;

For4, 5′-CTTCGCGCCACACTTGTTG-3′;

Rev4, 5′-AAAACCTGTTAGCCCCGATG-3′.

Primers to amplify DCV are provided in reference 19. Amplicons were pooled, purified with the NucleoSpin kit (MACHEREY-NAGEL), sheared in a Bioruptor Pico (Diagenode) in 1.5 mL Bioruptor tubes (Diagenode) to an average size of 200 bp following 20 cycles of 30 s of sonication and 30 s of cooling. The library was prepared using the NEB Next Ultra II DNA Library Prep kit (NEB) according to the manufacturer’s instructions and multiplexed with barcodes (E7335, E7500, E7710, E7730, NEB). Library size was determined using the DNA 1000 Bioanalyzer kit (Agilent), and the concentration was measured with Qubit dsDNA High Sensitivity assay kit (Thermo Fisher). Libraries were sequenced on an Illumina NextSeq200 as paired-end 55 bp long reads and demultiplexed with bcl2fastq (RRID:SCR_015058).

### NGS data processing

Raw Illumina read data were processed using the bioinformatics pipeline V-pipe 3.0 (55), which was integrated into a custom Snakemake workflow. The CrPV and DCV parental stock data sets were aligned to their respective reference sequences (CrPV, NC_003924.1 and DCV EB, NC_001834.1), using the Burrows-Wheeler Alignment Tool BWA-MEM (56). Consensus sequences were generated for both parental stock data sets using SmallGenomeUtilities (57). These consensus sequences were used as references for aligning raw reads from the samples at passages 1, 2, 3, 5, and 10. Sequencing errors were corrected and mutations called relative to the parental stock consensus sequence using the tool VILOCA (version 1.1.0). VILOCA achieves this by clustering sequencing reads into local haplotypes approximately as long as the read length through a finite Dirichlet process mixture model, which integrates read quality scores to reliably distinguish true variants from sequencing noise (58). Non-synonymous mutations were annotated using an adapted version of vcf_annotator (https://github.com/rpetit3/vcf-annotator). Population nucleotide diversity was calculated using SNPGenie (59), which estimates average pairwise nucleotide differences directly from the observed allele frequencies at each position after error correction. SNPGenie computes overall diversity and diversity per synonymous (piS) and non-synonymous (piN) sites, weighting each site by the fraction of possible substitutions that are synonymous or nonsynonymous. The minfreq parameter in SNPGenie was set to zero. The complete computational workflow and notebooks for generating figures are available on GitHub (https://github.com/cbg-ethz/DCV-CrPV-cGAS-STING-pathway-data-analysis).

### Mean nucleotide diversity

A linear mixed-effects model was fitted to analyze the log_10_-transformed DCV mean nucleotide diversity (lme, nlme package in R [60]). First, a model was constructed including passage, condition, and their interaction as fixed effects, along with evolutionary lineage replicate as a random effect to account for non-independence within replicates. To allow for heteroscedasticity between passages, a variance structure (VarIdent) was included, and since slight autocorrelation across passages within lineages was detected, an autoregressive moving average correlation structure (corARMA, q = 1) was incorporated. This model was then compared to a simpler model without the interaction term using ANOVA. Since ANOVA indicated no significant difference between the full and simplified models, the simpler model was used due to its fewer degrees of freedom. Residuals versus fitted values plots, as well as Levene’s tests for homogeneity of variances across passage, genotype, and their interaction, did not indicate significant heteroscedasticity. Q-Q plots of the residuals showed approximate normality.

A similar mixed-effects model was fitted to analyze the log_10_-transformed CrPV mean nucleotide diversity. To account for the piecewise linear structure of the data (initially increasing linearly, then decreasing), passage number was included as fixed effects along with an indicator variable for passage 10. This approach allowed modeling a potential change in the effect of passage after passage 5. An interaction term between condition and this indicator variable was also included to test for condition-specific changes after passage 10. Additionally, to account for within-lineage autocorrelation across passages, an AR(1) autocorrelation structure was incorporated in the model residuals using the corAR1 function from the nlme package. Model assumptions were confirmed using check_model from the performance package in R (61).

CrPV gene diversity was analyzed using ANOVA (R rstatix, anova_test [62]) with the dependent variable being the mean diversity over the passages for each replicate line, the between-subject factor being condition, and the within-subject factor being gene. Normality was confirmed using qq-plots, homogeneity of variances was confirmed using Levene’s test (R, levene_test), and homogeneity of covariances of the between-factors was tested using Box’s M-Test (R, box_m). As post hoc analysis, pairwise comparisons between all gene levels were performed using estimated marginal means with Bonferroni adjustment for multiple testing (emmeans package in R [63]).

### Nucleotide diversity in 2B and the remaining coding region in DCV and CrPV populations

Nucleotide diversity in the *2B* gene was compared to the remaining coding region by computing the mean diversity over the passages and then using a linear mixed-effects model (lme4 package in R [64]), with the dependent variable being the log-transformed diversity for each evolutionary lineage. The fixed effects in the model were virus (DCV or CrPV), condition (included as a control variable), and genome region, while the evolutionary lineage was included as a random effect. A sensitivity analysis confirmed that removing the condition from the model did not alter the significance or effect sizes of virus or genome region, supporting its exclusion. The normality of residuals was assessed using Q-Q plots and the Shapiro-Wilk test (shapiro.test). The homogeneity of variance was evaluated using plots of residuals versus fitted values and Levene’s test (car package in R). The independence of residuals was checked through plots of residuals and autocorrelation function (ACF) plots. Post hoc pairwise comparisons were performed using estimated marginal means with Tukey’s adjustment for multiple comparisons between virus and genome region (emmeans package in R).

### Statistical analysis for titers and relative RNA levels

A mixed ANOVA with a within-subject factor, passage, and an independent between-subject factor, host condition, was used to assess variation among log-transformed viral titers between conditions and passages. Subsequently, a pairwise comparison of host conditions at each passage level was performed using estimated marginal means (R package emmeans, v1.10.7 [63]), adjusting *P*-values with Bonferroni correction. ANOVA assumptions were confirmed using the R package *performance* (61) with the function check_homogeneity (Levene’s test, *P* > 0.05), check_sphericity (*P* > 0.05), and check_normality, where the points remained within the confidence interval, suggesting that the residuals are approximately normally distributed. Residual diagnostics, including Durbin-Watson tests (DCV: *P* = 0.998, CrPV: *P* = 0.07) and ACF plots of residuals, indicated no significant autocorrelation, supporting the use of a model without an autocorrelation structure. Nevertheless, given the temporal structure of the data, a model incorporating an AR(1) autocorrelation structure was also fitted as a sensitivity analysis. This alternative model exhibited some collinearity and some deviations from normality in residuals. However, the significance of *P*-values remained unchanged, suggesting that the main model inference is robust to potential autocorrelation. This analysis was performed for each virus separately.

To compare the relative RNA levels between WT and D29N CrPV, a linear mixed-effects model was used on the log-transformed values (lme, nlme package in R [60]). A model was fitted that included host condition, time post-infection, and variant (D29N or WT) as additive effects. To account for measurements from the same lineage over time, a random intercept was included for time post-infection within each lineage. Additionally, to model the correlation of the measurements over time post-infection within each lineage, a first-order autoregressive (AR(1)) correlation structure was incorporated. Model assumptions were confirmed using check_model from the performance package in R (61), revealing only mild deviations from the model assumptions. Residual autocorrelation was assessed using the ACF, confirming that AR(1) correlation structure successfully eliminated temporal dependencies.

### Approximation of the selection coefficient of the D29N mutation

Two complementary approaches were used to estimate the fitness effect of the D29N mutation: replication rate analysis and frequency data analysis. The replication rates (r) of the WT and mutant (D29N) were computed based on the fold change (x) representing RNA levels at 96 hours post-infection (hpi) relative to 0 hpi, assuming an exponential growth model:


$$x=e^{\frac{t}{10}r}$$

where t is time in hours and assuming a 10-hour generation time (65). Using the calculated replication rates, the selection coefficient ($s_{RNA}$) was determined by comparing the growth advantage of D29N relative to P0 over one generation (66):


$$S_{RNA}=\frac{r_{mutant}-r_{wildtype}}{r_{wildtype}}$$

The selection coefficient ($s_{freq}$) was also estimated using changes in D29N frequencies over time (67):


$$S_{freq}=\frac{\ln\;\left(\frac{f_{mutant}}{1-f_{mutant}}\right)\;final-\ln\;\left(\frac{f_{mutant}}{1-f_{mutant}}\right)\;initial}{n}$$

with *n* being the number of total generations. For this analysis, only evolutionary lineages with multiple time points in which D29N occurred were included, as single-time-point data do not allow for the determination of frequency change over time. Positive selection coefficients indicate that the mutant grows faster than the WT, while negative coefficients indicate slower growth.

### Protein structure prediction

AlphaFold 3 (27) was used to predict the CrPV 2B structure, which was simulated with the addition of oleic acid, myristic acid, palmitic acid, and Ca^2+^ as ligands and ions.

### 2B sequence logo and homology detection

The CrPV 2B protein sequence was used in a homology search using BLASTp (68) against the non-redundant databases (NRDB) with an E value cutoff of 0.05, resulting in 131 homologs that were mainly from *Riboviria*. A multiple alignment with all the obtained proteins was created on the NCBI site with Cobalt (69), and the first 50 amino acids of the 2B protein in the alignment were used to construct a sequence logo (70). Homology of the CrPV 2B protein with other proteins was established with PSI-BLAST (22) using default parameters (E < 0.005 for inclusion in the next iteration) against the NRDB database and iterating until convergence. To test the homology of CrPV 2B with poliovirus (Q1PHW2 in UniProt) and CVA10 (A0A6M2Z865 in UniProt) polyproteins, HHPred was used in the pairwise alignment mode using default parameters.

### Immunofluorescence assay

*Drosophila* S2 cells were seeded on 0.01% poly-D-lysine-coated coverslips in 24-well plates and incubated for 3 h at 27°C. Cells were then transfected with 0.5 µg of the indicated plasmids using 1 µL of X-tremeGENE HP (Roche), and the medium was refreshed after 24 h. For co-transfection of two plasmids, 0.25 µg of each plasmid was used instead. All following steps were performed at room temperature and in the dark when possible. At 48 h after transfection, cells were fixed in 4% paraformaldehyde for 15 min, permeabilized with 0.1% Triton-X100 in PBS for 15 min, and washed with PBS containing 0.1% Tween-20. Cells were blocked in 2% normal goat serum (NGS) for 30 min and incubated for 45 min with the primary antibody diluted in 2% normal goat serum in PBS. Calnexin 99A antibody (Developmental Studies Hybridoma Bank, Cnx99A 6-2-1; RRID:AB_2722011) was diluted 1:10, GM130 antibody (Abcam, ab30637) was diluted 1:250, Rab7 antibody (Developmental Studies Hybridoma Bank, RRID:AB_2722471) was diluted 1:10, and V5 antibody (Invitrogen, 46-0705) was diluted 1:200. After washing, cells were stained with secondary antibody goat anti-rabbit conjugated with Alexa Fluor 594 (Thermo Fisher, A11012) and goat anti-mouse with Alexa Fluor 647 (Thermo Fisher, A21235), used at a 1:500 dilution. Nuclei were stained with 1 µg/mL DAPI (Sigma) for 10 min, and coverslips were mounted on slides in ProLong Glass (Thermo Fisher). Cells were imaged on a Zeiss LSM900 Airyscan and 63× oil objective using the 405, 488, 561, and 633 excitation lasers. Images were Airyscan processed before analysis with Fiji software (71). Brightness and contrast were adjusted for visualization purposes. Raw images were used for colocalization analysis using the JACoP plug-in in Fiji (72). Images from individual cells were cropped, and the Pearson’s correlation coefficient between the eGFP and V5 signal was determined by applying the Costes automatic threshold. The coefficients were compared using a one-way ANOVA with Tukey’s multiple comparison test. Shapiro-Wilk normality test confirmed the normality of residuals and Levene’s test confirmed equal variances.

### Immunoprecipitation

Immunoprecipitation was performed following the instructions provided with the ChromoTek GFP-Trap Magnetic Agarose Kit (Proteintech). Briefly, S2R+ cells were co-transfected with plasmids encoding eGFP, 2B-eGFP, or 2B(D29N)-eGFP in combination with a plasmid encoding Sting-V5. Cells were rinsed with PBS and lysed in ice-cold lysis buffer (10 mM Tris-HCl, pH 7.5, 150 mM NaCl, 0.5 mM EDTA, 0.5% NP-40, and 1 mM PMSF). The cell lysates were centrifuged at 17,000 × *g* for 15 min at 4°C. The supernatants were diluted with lysis buffer, equilibrated GFP-Trap beads were added, and samples were incubated with end-over-end rotation for 1 h at 4°C. The beads were then washed four times with washing buffer (10 mM Tris-HCl pH 7.5, 150 mM NaCl, 0.5 mM EDTA, 0.05% NP40, and 1 mM PMSF) and resuspended in Laemmli sample buffer. The samples were heated for 5 min at 95°C, resolved on 12% SDS-PAGE gels, and transferred to nitrocellulose membranes by wet transfer. Membranes were stained with antibodies against GFP (rat, 1:1000, ChromoTek, 3h9-150), V5 tag (mouse, 1:2000, Invitrogen, 46-0705), or GAPDH (rabbit, 1:1000, Bioss, bs-8778R), followed by secondary antibodies IRDye 800CW goat anti-rat IgG (LICORBio, 926-32219), IRDye 800CW goat anti-mouse IgG (LICORBio, 926-32210), and IRDye 800CW goat anti-rabbit IgG (LICORBio, 926-32211), respectively. All secondary antibodies were used at a 1:2,000 dilution. The membranes were imaged using an Odyssey M Imaging System.

## Data Availability

All raw NGS data have been deposited in NCBI BioProject under the accession number PRJNA1257840. All other relevant data are within the article and its supplemental material.

## SUPPLEMENTAL MATERIAL

Supplemental material jvi.01239-25-s0001.pdf Figures S1 to S5; Tables S1 to S5.

ASM does not own the copyrights to Supplemental Material that may be linked to, or accessed through, an article. The authors have granted ASM a non-exclusive, world-wide license to publish the Supplemental Material files. Please contact the corresponding author directly for reuse.
