# Supplementary material for: A recurrent adaptive mutation in the transmembrane 2B protein of an insect picorna-like virus in a nonnative host
Source: J Virol. 2025 Dec 22;100(1):e01239-25. doi: 10.1128/jvi.01239-25 (PMC12817933; doi:10.1128/jvi.01239-25)
Supplement: Supplemental material — Figures S1 to S5; Tables S1 to S5. [file jvi.01239-25-s0001.pdf]

## Supplementary Material for:

# A recurrent adaptive mutation in the transmembrane 2B protein of an insect picorna-like virus in a nonnative host

Oscar M. Lezcano[1*], Lara Fuhrmann[2,3*], Reinder Bos[1], Haitao Wang[1,4], Milou Stevens[1], Niko Beerenwinkel[2,3], Martijn A. Huynen[5], Ronald P. van Rij[1]

1. Department of Medical Microbiology, Radboud University Medical Center, Nijmegen, 6500 HB, Nijmegen, the Netherlands.
2. Department of Biosystems Science and Engineering, ETH Zurich, Basel, 4056, Switzerland
3. SIB Swiss Institute of Bioinformatics, Basel, 4058, Switzerland.
4. Institute of Plant Protection, Key laboratory of Food Quality and Safety of Jiangsu Province, Jiangsu Academy of Agricultural Sciences, Nanjing 210014, China.
5. Department of Medical BioSciences, Radboud University Medical Center, 6500 HB, Nijmegen, the Netherlands.

*These authors contributed equally to this study.

**Corresponding authors:** niko.beerenwinkel@bsse.ethz.ch; martijn.huijnen@radboudumc.nl; ronald.vanrij@radboudumc.nl

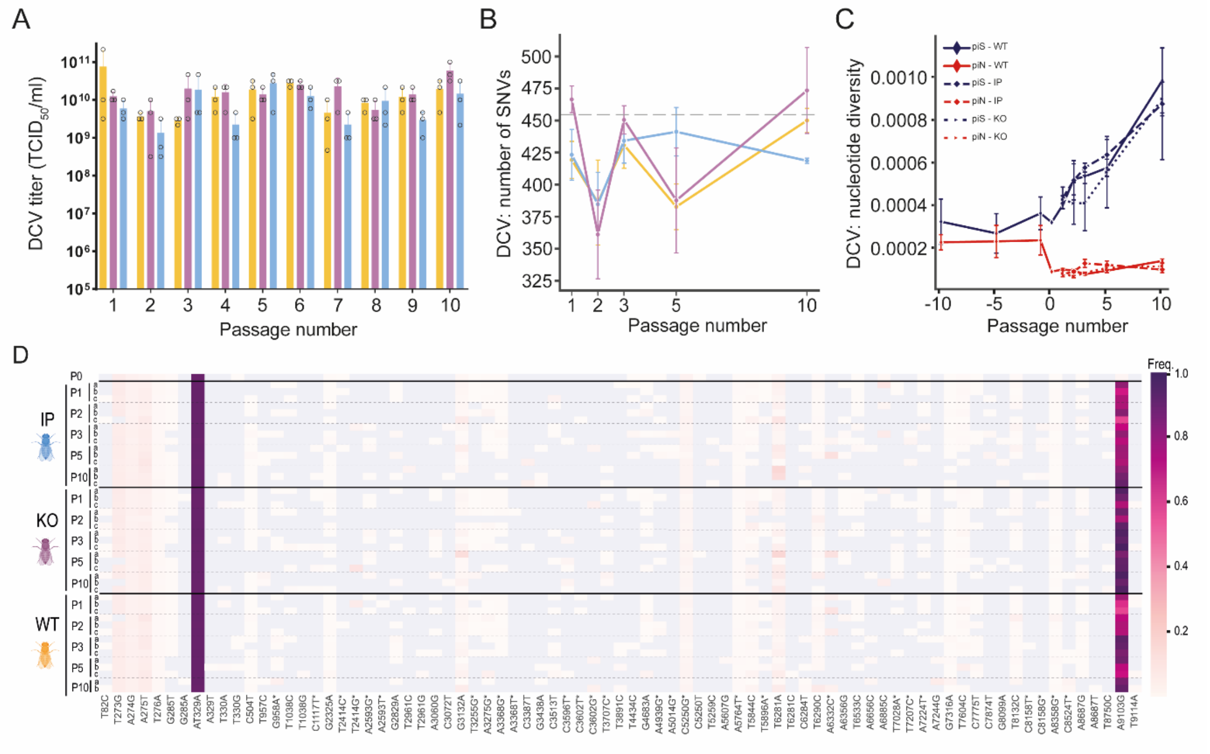


**Fig S1**. No evidence for adaptive evolution of DCV in its natural host, *Drosophila melanogaster*, with differing cGAS-STING response. (**A**) DCV titers in lysates of flies after each passage in the indicated host background. Color coding corresponds to Fig 1; yellow, WT; purple, *Sting* KO; blue, immune primed (IP). Bars represent the means and SD of the three replicate viral lineages; circles indicate individual data points. (**B**) The number of single-nucleotide variants (SNVs) in DCV populations from WT, *Sting* KO, and IP flies across passages. Data are shown as means and SEM across the three replicate lineages for each condition. The dashed line marks the number of SNVs in the parental stock. (**C**) Nucleotide diversity per synonymous (piS) and non-synonymous (piN) site of DCV populations across passages. Diversity values from DCV evolved in WT flies from our previous study (19) are integrated and denoted as passages -10, -5, and -1. Passage 0 is the parental stock, and passages 1 to 10 are the evolved DCV populations of the current study. Data are shown as means and standard errors across the three replicate lineages for each genotype. (**D**) Heatmap of SNV frequencies of all SNVs for DCV populations from WT, *Sting* KO and IP flies. Non-variable sites are indicated in gray. The *x*-axis represents genome positions relative to the DCV EB reference strain (NC_001834.1). Only SNVs that occurred in at least one sample with an observed frequency ≥ 0.1 are shown. Non-synonymous mutations are marked with an asterisk.

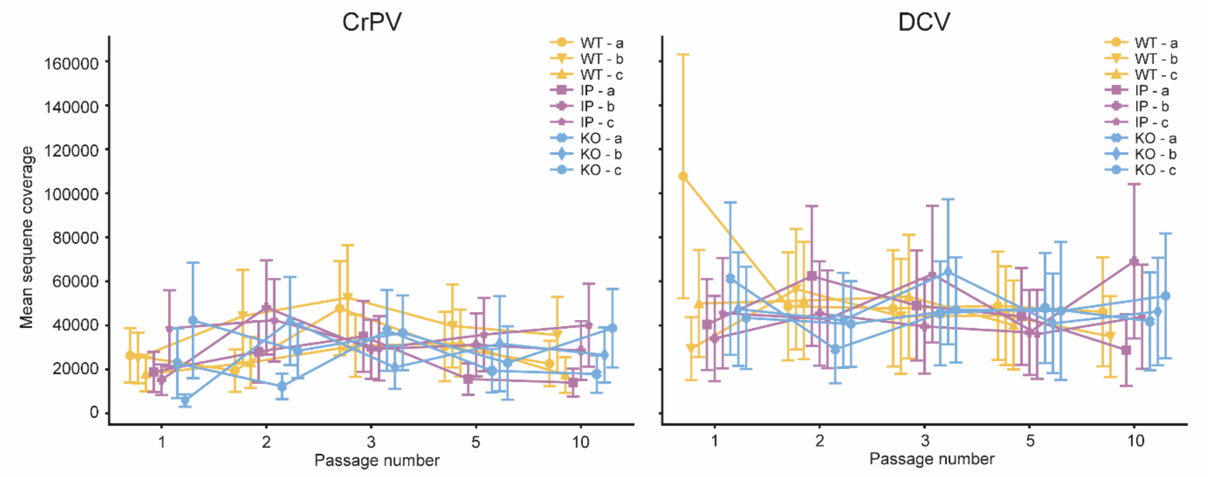


**Fig S2.** Sequence coverage across genome positions for CrPV and DCV samples. For each sample, the plotted value represents the average sequencing coverage across all positions in the viral genome. Error bars denote the standard deviation of coverage across these positions, illustrating the variability of read depth within each genome. Lowercase letters a, b, c indicate the three independent evolutionary lineages.

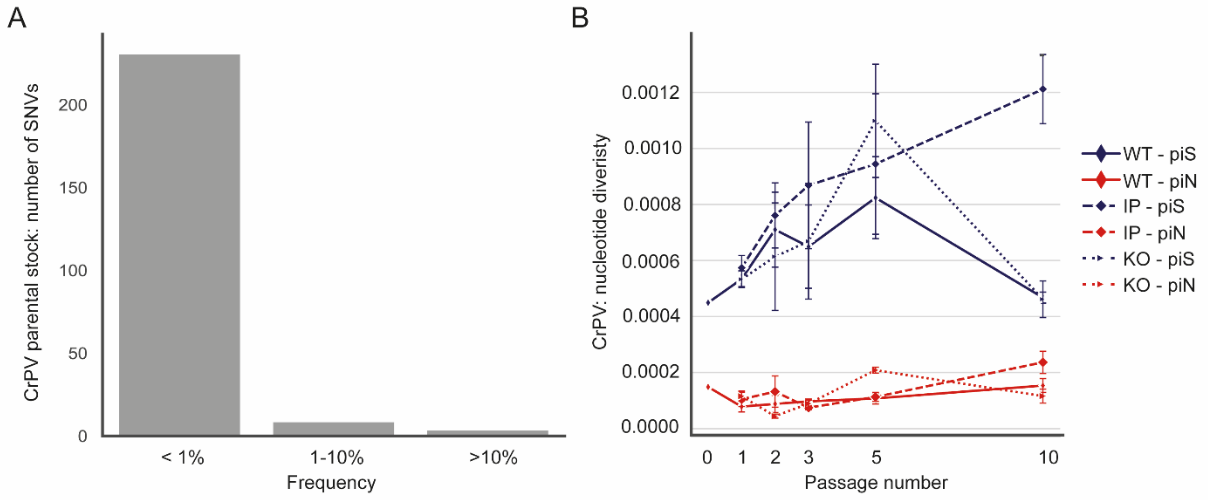


**Fig S3.** Single-nucleotide variants (SNVs) in the CrPV parental stock and nucleotide diversity in the evolutionary lineages. (**A**) Frequency distribution of SNVs in the indicated bins for the parental CrPV stock. (**B**) CrPV population nucleotide diversity per synonymous (piS) and non-synonymous (piN) site across passages. Data are shown as means and standard error across the three replicate lineages for each condition.

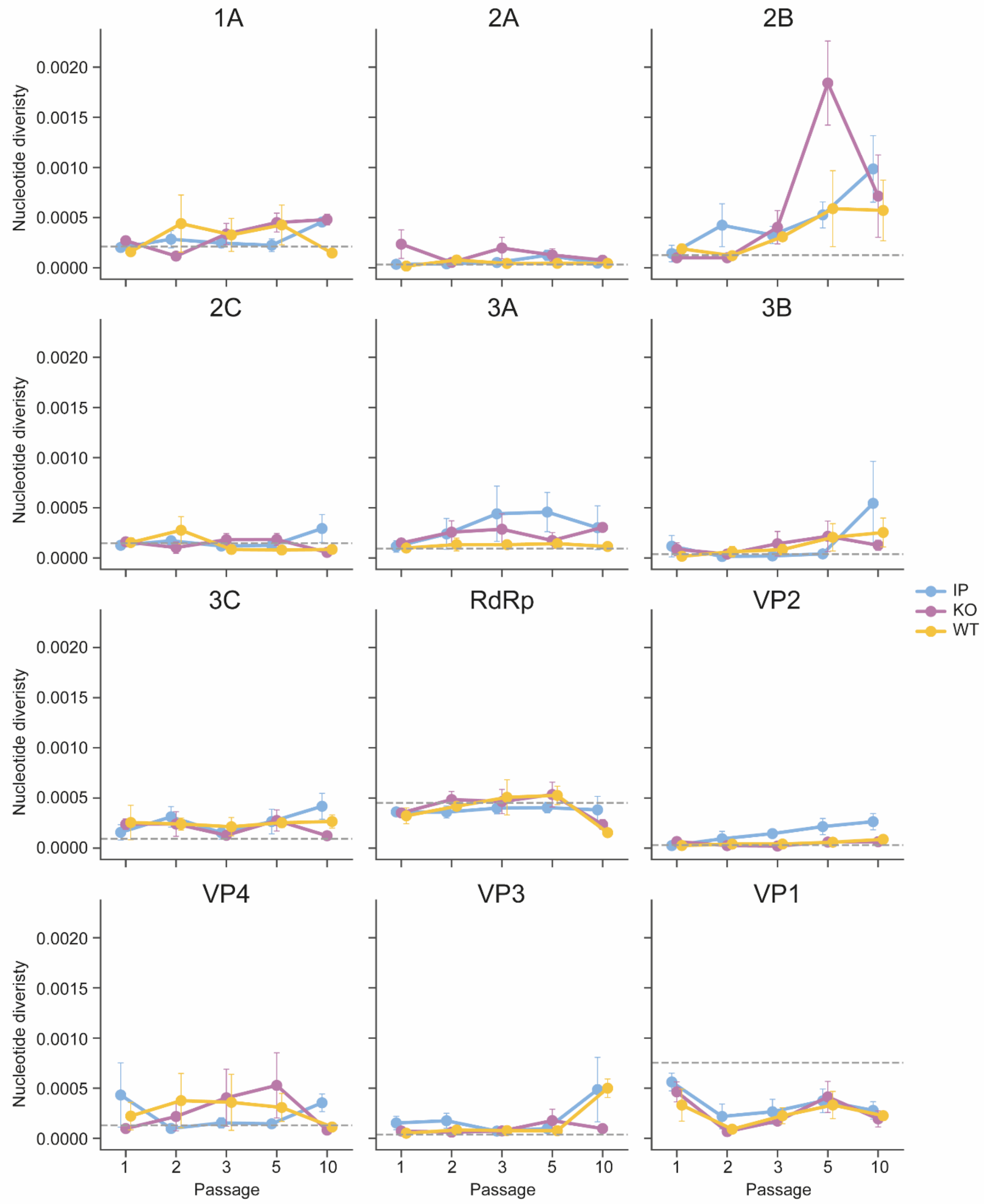


**Fig S4.** Temporal dynamics of mean nucleotide diversity per site of each CrPV gene. Data are shown as means and standard errors across the three replicate lineages for each host background.

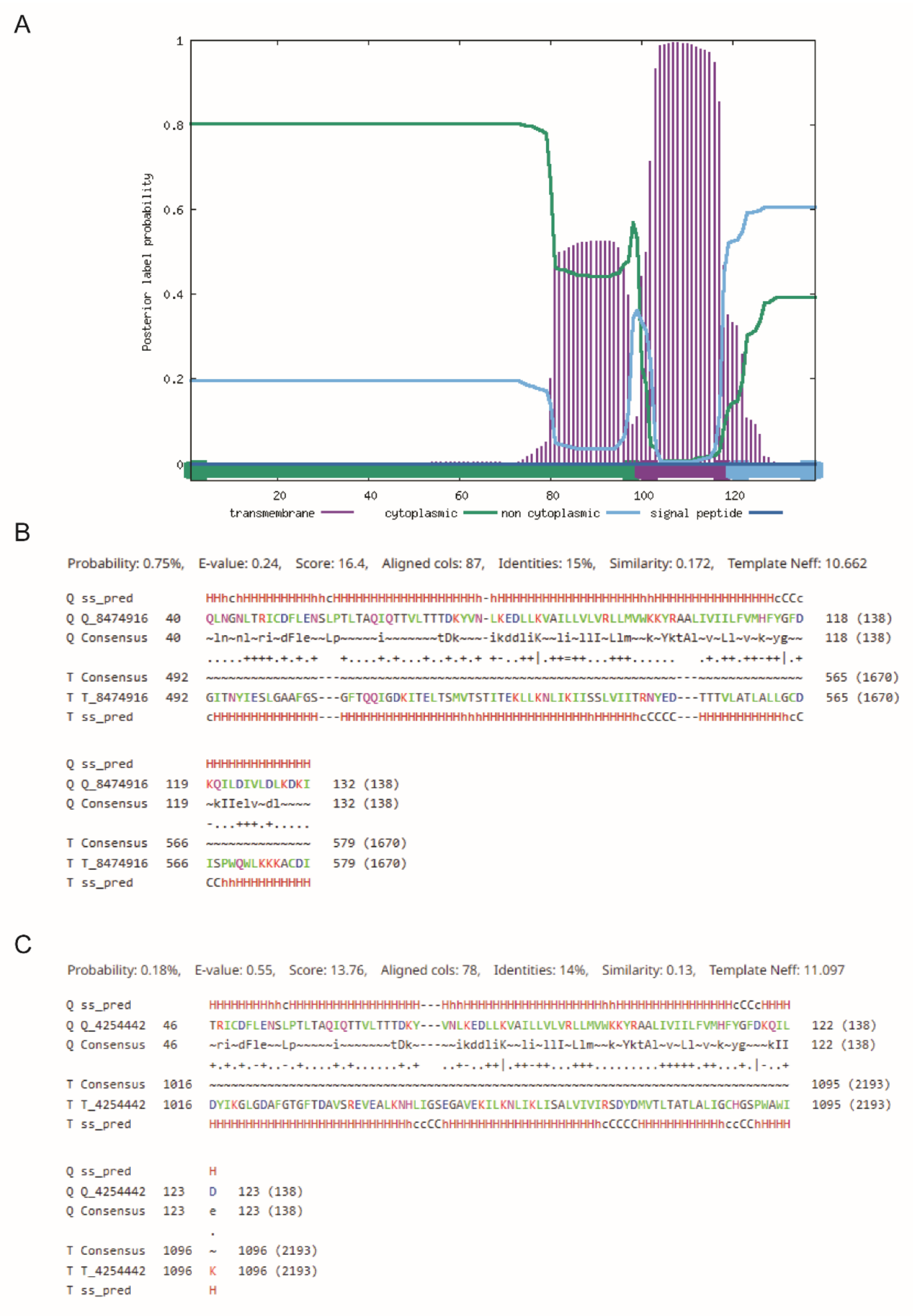


**Fig S5.** Structural predictions and homology of the CrPV 2B protein. (**A**) Transmembrane region prediction plot for CrPV 2B, created with PolyPhobius (Käll L et al, 2004. J Mol Biol 338:1027–1036; DOI: 10.1016/j.jmb.2004.03.016) using default parameters. (**B, C**) Alignment of the CrPV 2B protein (top, "Q", uniprot id: Q9IJX4) with the complete polyprotein of (B) poliovirus (bottom, "T", uniprot id: Q1PHW2) and (C) the coxsackievirus A10 (CVA10) (bottom, "T", uniprot id: A0A6M2Z865). In order to find support for the homology of CrPV 2B with enterovirus 2B, we ran HHpred in the pairwise alignment mode with default parameters. Among the polyproteins of poliovirus and CVA10 polyproteins, the 2B proteins (position 491–589 in poliovirus and position 1013–1111 in CVA10) are the most likely homologous region with CrPV 2B, even though the E-value is not significant. The top and bottom red letters indicate the predicted secondary structure per sequence (H, alpha helix; C, coil). Sequences of the viruses are indicated in multicolor font, and the consensus sequences are shown with symbols indicating moderately conserved ("+") and strongly conserved ("I") positions. The lack of a consensus sequence for the polio and coxsackievirus 2B proteins is caused by the low level of conservation of the 2B protein in both enteroviruses relative to the other enterovirus proteins.

**Table S1.** Summary of fixed and random effects from the linear mixed model predicting the log-transformed DCV nucleotide diversity based on host background (condition) and passage number as fixed effects and replicate lineage as a random effect.

| Effect type | Effect | Estimate | Std. Error | t-value | p-value |
|---|---|---|---|---|---|
| Fixed Effects | Intercept | -3.6350 | 0.02096 | -173.3920 | < 0.0001 |
| | Passage | 0.0156 | 0.0029 | 5.2617 | < 0.0001 |
| | Condition IP | -0.0026 | 0.0276 | -0.0963 | 0.9264 |
| | Condition KO | -0.0563 | 0.0276 | -2.0377 | 0.0877 |
| | | **Std. Dev.** | | | |
| Random Effect | Replicate (Intercept) | 0.0201 | | | |
| | Residual | 0.0308 | | | |

**Table S2.** Summary of fixed and random effects from the linear mixed model predicting the log-transformed CrPV nucleotide diversity based on host background (condition), passage number, passage 10 as a separate binary factor, and an interaction term with fly condition and passage 10 as fixed effects and replicate as a random effect.

| Effect type | Effect | Estimate | Std. Error | t-value | p-value |
|---|---|---|---|---|---|
| Fixed Effects | Intercept | -3.77 | 0.048 | -78.180 | < 0.001 |
| | ConditionIP | -0.003 | 0.05 | -0.06 | 0.9544 |
| | ConditionKO | -0.001 | 0.05 | -0.023 | 0.982 |
| | Passage | 0.046 | 0.012 | 3.920 | < 0.001 |
| | Passage10 (binary indicator) | -0.391 | 0.107 | -3.645 | < 0.001 |
| | Condition IP: Passage10 | 0.245 | 0.095 | 2.592 | 0.014 |
| | Condition KO: Passage10 | -0.028 | 0.095 | -0.294 | 0.771 |
| | | **Std. Dev** | | | |
| Random Effect | Replicate (Intercept) | 0.027 | | | |
| | Residual | 0.102 | | | |

**Table S3.** Results of post-hoc pairwise comparisons of mean diversity between CrPV genes across passages. Pairwise contrasts were performed using estimated marginal means with Bonferroni adjustment for multiple comparisons. Only significant contrasts are shown.

| | Estimate | SE | df | t-ratio | p-value |
|---|---|---|---|---|---|
| 2A - 2B | −4.085e-4 | 3.989e-05 | 22 | -10.244 | <.0001 |
| 2B - VP2 | 4.07e-4 | 3.989e-05 | 22 | 10.203 | <.0001 |
| 2B - 3B | 3.580e-4 | 3.989e-05 | 22 | 8.977 | <.0001 |
| 2B - 3B | 3.580 e-4 | 3.989e-05 | 22 | 8.977 | <.0001 |
| 2B - 2C | 3.425e-4 | 3.989e-05 | 22 | 8.586 | <.0001 |
| 2B - VP3 | 3.389e-4 | 3.989e-05 | 22 | 8.497 | <.0001 |
| 2A - RdRp | -3.128e-4 | 3.989e-05 | 22 | -7.842 | <.0001 |
| RdRp - VP2 | 3.111e-4 | 3.989e-05 | 22 | 7.802 | <.0001 |
| 2B - 3A | 2.662e-4 | 3.989e-05 | 22 | 6.673 | 0.0001 |
| 3B - RdRp | -2.623e-4 | 3.989e-05 | 22 | -6.575 | 0.0001 |
| 2B - 3C | 2.532e-4 | 3.989e-05 | 22 | 6.347 | 0.0001 |
| 2C - RdRp | -2.467e-4 | 3.989e-05 | 22 | -6.185 | 0.0002 |
| RdRp - VP3 | 2.431e-4 | 3.989e-05 | 22 | 6.096 | 0.0002 |
| 2B - VP4 | 2.298e-4 | 3.989e-05 | 22 | 5.761 | 0.0004 |
| 1A - 2A | 2.238e-4 | 3.989e-05 | 22 | 5.612 | 0.0006 |
| 1A - VP2 | 2.222e-4 | 3.989e-05 | 22 | 5.571 | 0.0006 |
| 2B - VP1 | 2.084 e-4 | 3.989e-05 | 22 | 5.224 | 0.0014 |
| 2A - VP1 | -2.002e-4 | 3.989e-05 | 22 | -5.020 | 0.0023 |
| VP2 - VP1 | -1.986e-4 | 3.989e-05 | 22 | -4.979 | 0.0025 |
| 1A - 2B | -1.848e-4 | 3.989e-05 | 22 | -4.632 | 0.0056 |
| 2A - VP4 | -1.788e-4 | 3.989e-05 | 22 | -4.483 | 0.0078 |
| VP2 - VP4 | -1.772e-4 | 3.989e-05 | 22 | -4.443 | 0.0086 |
| 1A - 3B | 1.733e-4 | 3.989e-05 | 22 | 4.344 | 0.0107 |
| 3A - RdRp | -1.704e-4 | 3.989e-05 | 22 | -4.271 | 0.0126 |
| 1A - 2C | 1.577e-4 | 3.989e-05 | 22 | 3.954 | 0.0254 |
| 3C - RdRp | -1.574e-4 | 3.989e-05 | 22 | -3.945 | 0.0259 |
| 2A - 3C | -1.554 e-4 | 3.989e-05 | 22 | -3.897 | 0.0288 |
| 1A - VP3 | 1.542e-4 | 3.989e-05 | 22 | 3.865 | 0.0308 |
| 3C - VP2 | 1.538e-4 | 3.989e-05 | 22 | 3.856 | 0.0314 |
| 3B - VP1 | -1.497e-4 | 3.989e-05 | 22 | -3.753 | 0.0392 |

**Table S4.** Summary of the linear mixed model predicting the log-transformed viral loads of CrPV wildtype and D29N mutant.

| **Effect** | **Estimate** | **Std. Error** | **t-value** | **p-value** |
|---|---|---|---|---|
| Intercept | -8.768 | 0.457 | -19.175 | < 0.0001 |
| Time post infection | 0.14 | 0.006 | 22.925 | < 0.0001 |
| Variant D29N | 2.632 | 0.382 | 6.886 | < 0.0001 |
| Condition IP | -1.856 | 0.468 | -3.964 | 0.0014 |
| Condition KO | 0.000 | 0.468 | 0.000 | 0.999 |

**Table S5.** Estimates of D29N selection coefficients based on mutation frequency changes ($s_{freq}$) and CrPV RNA levels ($s_{RNA}$).

| **Estimation based on mutation frequency changes** | | | |
|---|---|---|---|
| **Evolved lineage** | $s_{freq}$ | **mean** | |
| KOa | 0.312 | 0.287 | |
| WTa | 0.225 | | |
| WTc | 0.325 | | |
| **Estimation based on CrPV RNA levels** | | | |
| **Sample** | $s_{RNA}$ | **Mean** | **Mean** (all samples) |
| IP-1 | 0.161 | 0.328 | 0.267 |
| IP-2 | 0.568 | | |
| IP-3 | 0.256 | | |
| KO-1 | 0.506 | 0.375 | |
| KO-2 | 0.296 | | |
| KO-3 | 0.322 | | |
| WT-1 | 0.23 | 0.097 | |
| WT-2 | 0.107 | | |
| WT-3 | -0.047 | | |
